# The Influence of Functional Composite Coatings on the Properties of Polyester Films before and after Accelerated UV Aging

**DOI:** 10.3390/ma17133048

**Published:** 2024-06-21

**Authors:** Małgorzata Mizielińska, Magdalena Zdanowicz, Alicja Tarnowiecka-Kuca, Artur Bartkowiak

**Affiliations:** Center of Bioimmobilisation and Innovative Packaging Materials, Faculty of Food Sciences and Fisheries, West Pomeranian University of Technology Szczecin, Janickiego 35, 71-270 Szczecin, Poland; mmizielinska@zut.edu.pl (M.M.); mzdanowicz@zut.edu.pl (M.Z.); alicja.tarnowiecka-kuca@zut.edu.pl (A.T.-K.)

**Keywords:** active packaging, active coatings, *Achillea millefolium* L., *Hippophae rhamnoides* L., herbal tracts, *Hypericum* L., phi6 phage, polyester films, poly(hydroxybutyrate-co-hydroxyvalerate), poly(lactic acid), zinc oxide

## Abstract

The aim of this study was to cover biopolymeric packaging films based on PLA/PHBV blend with a functional composite coating (to retain their ecological character) and to investigate their antimicrobial properties before and after UV irradiation. As an active coating, the carrier hydroxypropyl methyl cellulose (HPMC), as well as its modified form with *Achillea millefolium* L., *Hippophae rhamnoides* L., and *Hypericum* L. extract (E) and a combined system based on the extracts and nano-ZnO (EZ), was used to obtain active formulations. Additionally, film surface morphology (SEM, FTIR-ATR) and color (CIELab scale) analysis of the pre- and post-UV-treatment samples were performed. The results confirmed that the E and EZ-modified films exhibited antibacterial properties, but they were not effective against phage phi6. Q-SUN irradiation led to a decrease in the activity of E coating against *Staphylococcus aureus*, *Pseudomonas syringae*, and *Candida albicans*. In this case, the effectiveness of EZ against *C. albicans* at 24 h and 72 h UV irradiation decreased. However, the irradiation boosted the antiviral effectiveness of the EZ layer. SEM micrographs of the film surface showed that UV treatment did not significantly influence the native film morphology, but it had an impact on the coated film. FTIR analysis results showed that the coatings based on HPMC altered the IR absorption of the nonpolar groups of the biopolyester material. The applied coatings only marginally affected film color changes and increased their yellowness after UV irradiation, whereas a composite layer of nano-ZnO limited these changes.

## 1. Introduction

In times of growing environmental concerns, alternative eco-friendly packaging materials should be developed, especially for short-term storage of food products. In this context, biopolymers that are biodegradable and bio-based such as poly(hydroxybutyrate-co-hydroxyvalerate) (PHBV), poly(lactic acid) (PLA) and their blends may be considered a promising alternative for oil-based plastics [1,2,3]. Furthermore, properly designed active biopolymeric (e.g., antimicrobial) materials may affect the shelf life of food products and limit food waste. Active additives can be introduced into the polymer matrix and processed with the polymer. However, due to shearing forces and high temperatures, the functionality of these additives can be influenced, especially when they consist of natural extracts or essential oils. To overcome this disadvantage, a coating of food packaging with an active formulation can be used. Such coatings may be applied not only to typical commercial nonbiodegradable films (e.g., PE, PP, PET) but also to biodegradable blends to make their surface active [4,5,6,7]. Selected plant extracts have demonstrated antibacterial and antiviral properties, which can be introduced into a hydrophilic coating carrier such as hydroxypropyl methyl cellulose (HPMC) to form functional coatings. Natural-origin HPMC is partly O-methylated and O-(2-hydroxy propylated) cellulose derivative. Due to a low amount of hydrophobic functional groups, the biopolymer is water soluble. This is why this carrier is often used as a film binder or a coating agent/carrier alone. Moreover, it is nontoxic and thermally and mechanically stable. Additionally, HPMC was confirmed to be an effective emulsifier. Its amphiphilic character enables it to act as a bridge between the oil and water phases, to form stable emulsion. It may be suggested that the introduction of an emulsifier to the HPMC (with hydrophilic antimicrobial agents such as plant extracts) can improve/facilitate the adhesion of the coating to the hydrophobic biopolymer’s/blend’s surface [4,5]. It is worth underlining that materials modified with plant extracts rich in active compounds (e.g., antimicrobial and antioxidant) lead to the prolongation of food products’ shelf life. It is important that extracts introduced as the layers’ component do not influence the biopolymer matrix’s physicochemical properties and biodegradability. Nevertheless, a high concentration of the herb extract that is dark green or even dark brown may form a nontransparent coloring layer [6,7].There is a need to mention that the coating’s transparency depends on the layer grammage/thickness. When the amount of active agent is reduced, the coating can become nearly transparent; however, its activity cannot always be maintained. This is why the formation of composite coating material with less concentrated herb extracts that have a synergistic antimicrobial effect in combination with ZnO nanoparticles [8] may be a solution.

*Achillea millefolium* L., sea buckthorn (*Hippophae rhamnoides* L.), and *Hypericum* species demonstrate multiple beneficial effects, including anti-inflammatory, antioxidant, diaphoretic, spasmolytic, choleretic, hepatoprotective, antipyretic, analgesic, anticancer, and antimicrobial properties [9,10,11,12]. The extracts of *A. millefolium* L. flower are rich in flavonoid glycosides, flavonoids, and some other phenolic compounds [10]. Many extracts of *A. millefolium* L. aerial parts were confirmed to be active toward bacterial, yeast, and mold strains such as *S. aureus*, *B. cereus*, *E. coli*, *P. aeruginosa*, *Klebsiella pneumoniae*, *Yersinia enterocolitica*, *Salmonella enteritidis*, *Aspergillus niger*, and *Candida albicans*. Additionally, the Gram-positive bacteria were found to be more susceptible than the Gram-negative ones [9,10,11,12]. Sea buckthorn is rich in flavonoids, procyanidins, tocopherols, and phytosterols [13]. The *H. rhamnoides* L. extracts were reported to have antimicrobial activity against *B. cereus*, *B. pumilus*, *E. coli*, *P. aeruginosa*, *S. aureus*, and *Enterococcus faecalis*, and *C. albicans* [14,15,16]. On the other hand, ethanolic extracts of *Hypericum* were evidenced to have high activity against *B. cereus*, *E. coli*, *S. aureus*, and *P. aeruginosa* [17,18]. Summarizing, extracts of the *Achillea millefolium* L., *Hippophae rhamnoides* L., and of *Hypericum* species were confirmed to have antimicrobial properties. It was assumed that the solution of extracts could be introduced into the biopolymer coating carrier to obtain active coating, which can be applied on biopolyester blend film to produce fully biodegradable packaging material.

In general, active layer-coated packaging should maintain its functionality during its own prolonged storage as well as during packed food product storage. Packaging materials with an internal coating should inhibit bacterial, yeast, and mold growth to extend the shelf life of the food products during the whole supply chain and food storage. Additionally, packaging materials with an external coating should also be active during all stages of a supply chain and storage to limit the spread of some viral particles (e.g., SARS-CoV-2) via human hands. This means that coatings should offer sufficient ultraviolet (UV) radiation barrier or be shielded against accelerated UV aging. The introduction of an active additive (to the coating carrier) that is resistant to UV, or a compound with shielding properties, can prevent an inactivation of the layer/coating after UV irradiation. It was confirmed that the application of nano-ZnO may improve the UV shielding of any packaging materials, i.e., biopolyesters or their blends. The ZnO nanoparticles are considered safe [19]. They can help maintain the antibacterial and antiviral properties of the active coatings [20,21,22,23,24].

The purpose of this work was to obtain active composite coating compositions based on HPMC with the presence of three selected herb extracts—*Achillea millefolium* L., *Hippophae rhamnoides* L., and *Hypericum* L. extract—and nano-ZnO filler and to apply them onto the surface of a composite PLA/PHBV film. The antimicrobial properties of the coated packaging material before and after UV treatment were studied. Additionally, an investigation of the coatings’ presence on the polyester blend morphology (SEM and FTIR-ATR analysis) and color changes (CIELab scale) was conducted.

## 2. Materials and Methods

### 2.1. Materials

The bacterial strains and phi6 phage used to determine antimicrobial properties were purchased from a collection from the Leibniz Institute Deutsche Sammlung von Mikroorganismen und Zellkulturen (DSMZ, Braunschweig, Germany). The bacterial strains used in this research were as follows: *B. subtilis* DSMZ 1090, *S. aureus* DSMZ 346, *E. coli* DSMZ 498, and *P. syringae* van Hall 1902 DSM 21482. The *P. syringae* strain was also used as a bacteriophage (Φ6) host. Phage phi 6 DSM-21518 was used as a SARS-CoV-2 surrogate to investigate the antiviral activity of the coatings. The yeast strain *C. albicans* PCM 2566 was purchased from the Polish Collection of Microorganisms.

Ethanol (98%, Warchem, Trakt Brzeski, Poland) was used to obtain herb extracts. The PLA/PHBV foil (20 μm) at the ratio 80:20 was obtained from PLA (Ingeo 4043D NatureWorks, Blair, NE, USA) and PHBV (Ecomann, EM 5400, Shenzhen Ecomann Biotechnology Co., Ltd., Guangdong, China). Hydroxypropyl-methyl cellulose (HPMC, Dow Wolff Cellulosics GmbH, Bomlitz, Deutschland) was used as a coating carrier in the experiments. Tween 20 (Pol-Aura, Szczecin, Poland) was used as an emulsifier. *Achillea millefolium* L., *Hippophae rhamnoides* L. (Flos, Mokrsko, Poland), and *Hypericum* L. (Kawon, Gostyń, Poland) were applied to prepare herb extracts. The zinc oxide AA 44899, (particle size diameter ~70 nm) powder (Thermo Fisher GmbH, Kandel, Germany) was used as an antimicrobial agent. To investigate the antimicrobial properties/activity of the coatings, MacConkey agar, TSA, Luria-Bertani (LB), and TSB broths (Merck, Darmstadt, Germany) were used. All the media were prepared in accordance with manufacturer protocols (all media were weighed according to Merck instructions, suspended in 1 L of distilled water, and autoclaved at 121 °C for 15 min).

### 2.2. Methods

#### 2.2.1. PLA/PHBV Blend Films’ Extrusion

A blend of the biopolyesters—PLA (Ingeo 4060D NatureWorks, Blair, Nebraska, USA) and PHBV (Ecomann, EM, Shenzhen Ecomann Biotechnology Co., Ltd., Guangdong, China)—at wt ratio 80:20 was prepared using a corotating twin-screw extruder (L/D = 40, d = 20 mm, Labtech Engineering Co., Ltd., Phraeksa, Thailand) at the temperature profile of 170/185/200 × 7/195 °C and rotational screw of 80 rpm. Then, the regranulate was transformed into a thin film using cast extrusion (L/D = 30, as above) at a single-screw extruder’s temperature profile of 185/190/195/195 °C. The width of the film was 170 mm and the thickness was 38–45 µm.

#### 2.2.2. Extracts Preparation

Dry herbs—*Achillea millefolium* L., *Hippophae rhamnoides* L., and *Hypericum* L.—were used to prepare the extracts. The dry plants were added individually into 100 mL of the 70/30% ethanol/water. The dispersions of the herbs were then added into sealed glass bottles and microwaved (Amica, Wronki, Poland) for 10 min at 70 °C. As a next step, the bottles were placed in a shaker (Ika, Staufen im Breisgau, Germany) and extracted for 1 h at 70 °C (150 rpm). The extraction was carried out according to the methods already reported by the authors [7,25,26] with a slight modification. After the extraction process, the herbs were separated from the extracts using a Büchner funnel and then filtered through a 0.2 μm filter and evaporated to obtain extract aqueous solutions with the following dry masses: 34.96% (*A. millefolium* L.), 33.5% (*H. rhamnoides*) and 35.03% (*Hypericum* L.).

#### 2.2.3. Preliminary Tests

A preliminary microbiological analysis was performed to indicate which extract was the most effective against model/reference Gram-positive and Gram-negative bacterial strains, such as *S. aureus* and *E. coli*.

The *S. aureus* strain was pre-grown on TSA agar, but *E. coli* cells were pre-grown on MacConkey agar (for 24 h, at 37 °C). Later, cell inoculums of 1.5 × 10^8^ CFU/mL of both strains (separately) were prepared in a sterile 0.85% NaCl solution. Then, 1 g of the 34.96% *A. millefolium* L. (% of dry mass), 1 g of the 33.5% *H. rhamnoides* (% of dry mass), and 1 g of the 35.03% *Hypericum* L. extract (% of dry mass) were added into a falcon test tube (wt ratio 1:1:1) to obtain a 3 g solution of the mixed herbal extract (S). As a next step, 1 g of each individual herb extract and 1 g of the solution S were added (separately) into the 9 g of LB. The samples were mixed with a vortex stirrer (150 rpm, Ika, Legnica, Poland) for 30 s. A prepared biomass of *S. aureus* or *E. coli* (100 μL) was added to the test tubes containing LB medium with the extracts (separately) or with the extract solution. The test tubes were introduced into BioSan bioreactors (BS-010160-A04, BioSan, Riga, Latvia) and incubated at 37 °C. The results of the preliminary analysis are included in the Supplementary Information (SI) section.

#### 2.2.4. Preparation of the Coating Formulation and Application onto PLA/PHBV Film Surface

**Figure 1:**
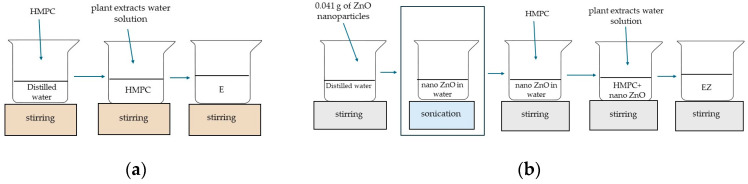
The steps of coating carrier preparation: (**a**) HMPC with plant extracts solution; (**b**) HMPC with nano-ZnO and plant extracts solution.

The individual extracts of *A. millefolium* L., *H. rhamnoides*, and *Hypericum* L. were added into a beaker at a wt ratio of 1:1:1. This meant that 10 g of the 34.96% *A. millefolium* L. extract, 10 g of the 33.5% *H. rhamnoides* extract, and 10 g of the 35.03% *Hypericum* L extract were mixed together to obtain a solution of herbal extracts (S). Then, 4 g of HPMC was introduced into 89 mL of distilled water (Figure 1a) and mixed for 1 h using a magnetic stirrer (Ika, Warsaw, Poland) at 1500 rpm at RT. When HPMC was fully dissolved, 1 mL of Tween 20 was added to the HPMC as an emulsifier. Then, 90 g of the carrier with the emulsifier was mixed with 10 g of *A. millefolium* L., *H. rhamnoides*, and *Hypericum* L. solution (S) and homogenized (1000 rpm, RT) (Heidolph, Sigma-Aldrich, Poznań, Poland). The 10 wt% herb aqueous extract solution with HPMC was thus obtained (E). As a next step, 0.041 g of nano-ZnO was added into distilled water (89 mL) (Figure 1b). Initially, the dispersion was mixed for 1 h using a magnetic stirrer (500 rpm, RT) and then sonicated for 30 min (amplitude: 20%, cycle: 0.5). After sonication, 4 g of the carrier was added into the dispersion and the system was mixed for 1 h using a magnetic stirrer (Ika, Warsaw, Poland) at 1500 rpm. After mixing, 1 mL of Tween 20 was added to the coating carrier. Then, 90 g of the whole was mixed with 10 g of the extract solution (S) and homogenized (1000 rpm) (Heidolph, Sigma-Aldrich, Poznań, Poland). The 10 wt% herb extract solution with nano-ZnO in HPMC was then obtained (EZ).

The polyester film was coated using the Unicoater 409 (Erichsen, Hemer, Germany) at ambient conditions with 12 μm diameter bars. Then, the samples were dried for 10 min at a temperature of 40 °C. Grammage values for the coatings on the film were 4.7 g/m^2^ and 6.8 g/m^2^ for E and EZ, respectively. Unmodified blend film was used as a control sample (C). The control and modified foil samples were cut into square shapes (3 cm × 3 cm).

#### 2.2.5. Q-SUN Irradiation

The uncoated film, as well as the film covered with E and EZ layers, was irradiated for 24 h (acronyms E24, EZ24) and 72 h (acronyms E72, EZ72) in a Q-SUN accelerated Xenon Test Chamber with 1.5 W/m^2^ (Model Xe-2, Q-LAB DEUTSCHLAND GMBH, Saarbrucken, Germany) as follows: at 60 °C; at 47 °C (black panel); and at 39 °C (chamber air) with RH of 40%.

#### 2.2.6. Antimicrobial Analysis

The antimicrobial activity of the covered and noncovered films was investigated based on the ASTM E 2180-01 standard [27]. To analyze the antiviral properties of the active layers, the bacteriophage Φ6 was purified according to a method described by Bhetwal et al. [28]. Then, the Φ6 lysate was prepared according to the method presented by Bonilla et al. [29]. The antiviral activity of the covered materials was compared with the noncovered biopolymer film and investigated according to the modified ISO 22196-2011 standard [30]. Finally, Φ6 particle amplification was performed as reported by Skaradzińska et al. [31]. The investigation of the *P. syringae* cultivation rate in real time with Φ6 lysate (after its contact/incubation with the PLA/PHBV film, and E and EZ layers, which meant that Φ6 lysate was incubated with the E and/or EZ coatings and/or control, uncoated film, separately) was carried out according to the ISO 22196-2011 standard [30]. Then, the Φ6 lysates were amplified in the host bacteria using BioSan bioreactors (BS-010160-A04, BioSan, Riga, Latvia) according to the method described in previous studies [7,8].

A statistical analysis of the outcomes from antimicrobial tests was carried out using analysis of variance (one-way ANOVA). Statistical significance was noted when *p* < 0.05. The significance was evaluated by using GraphPad Prism 8 (GraphPad Software, San Diego, CA, USA).

#### 2.2.7. SEM Analysis

Samples were tested using a scanning electron Vega 3 LMU microscope (Tescan, Brno-Kohoutovice, Czech Republic). As a first step, the samples before and after UV treatment (for 24 h and 72 h, respectively) were placed on pin stubs and coated with a thin layer of gold in a sputter coater at 24 °C (Quorum Technologies Q150R S, Laughton, East Sussex, UK). A SEM analysis was performed through the use of a tungsten filament with an accelerating voltage of 10 kV.

#### 2.2.8. FTIR-ATR Analysis

Fourier transform infrared (FTIR) spectrum with ATR transformation of both sides of the films (unmodified and coated) pre- and post-UV treatment was measured using an FTIR spectrophotometer (Perkin Elmer Spectrophotometer, Spectrum 100, Waltham, MA, USA), operated at a resolution of 4 cm^−1^ and with 16 scans. The spectrum was recorded at a wavelength of 4000–600 cm^−1^.

#### 2.2.9. Color Measurement of the Films

A CIELab color scale was analyzed using Colorimeter CR-5 (Konica Minolta, Tokyo, Japan). Each film was measured at 6 random locations. Moreover, changes in color (∆*E*), yellowness index (*YI*) [32], and chroma (c*), were calculated according to the following equations [33]:

$$\Delta E=\sqrt{\left[{\left(∆L*\right)}^{2}+{\left(∆a*\right)}^{2}+{\left(∆b*\right)}^{2}\right]}$$

*YI* = (142.86b*)/*L*

$$(c*)=\sqrt{\left[{\left(a*\right)}^{2}+{\left(b*\right)}^{2}\right]}$$

## 3. Results and Discussion

### 3.1. Antimicrobial Properties Analysis

A preliminary study (presented in the Supplementary Information Figure S1a,b) demonstrated that the solution of three extracts was active against *S. aureus* and *E. coli*. confirming their synergistic effect.

**Figure 2:**
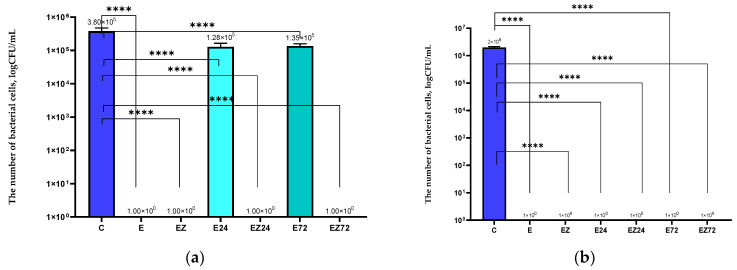
The influence of coatings on: (**a**) *S. aureus*, (**b**) *E. coli*; C—uncoated film; E—film coated with the HPMC layer with *Achillea millefolium* L., *Hippophae rhamnoides* L., and *Hypericum* L. extracts; EZ—film coated with the HPMC layer containing with *Achillea millefolium* L., *Hippophae rhamnoides* L. and *Hypericum* L. extracts and nanoparticles; E24, E72—film coated with E active layer after 24 h and 72 h of Q-SUN irradiation; EZ24, EZ72—film coated with EZ active layer after 24 h and 72 h of Q-SUN irradiation. One-way ANOVA; ****—*p* < −0.0001.

The results confirmed that the E coating based on the extract of the *Achillea millefolium* L., *Hippophae rhamnoides* L., and *Hypericum* L. inhibited *S. aureus* and *E. coli* growth completely (Figure 2a,b). The antimicrobial activity of the coating with the sea buckthorn was confirmed by Ong et. al. [34] and Brobbey et. al. [35]. These authors noted that phenolic compounds present in these plant extracts exhibited antimicrobial properties. The effectiveness of the EZ coating that contained the herbal extracts, together with an addition of the ZnO nanoparticles (toward *S. aureus* and *E. coli*), was also observed. Similar results were noted in previous research [6,8]. The active layers containing *Uncaria tomentosa* and *Formitopsis betulina* extracts or geraniol or carvacrol with the addition of nanoparticles of ZnO were effective against *S. aureus* and *E. coli*. Moreover, the synergistic effect between plant extracts and nano-ZnO was confirmed [6,7,8].

In our research, it was found that 24 h and 72 h of UV irradiation led to a decrease in the activity of E-coated samples against *S. aureus*. Similar results were obtained in a previous study [36] where a decrease in the effectiveness of the coating with *Eucomis comosa* extract against *S. aureus* after Q-SUN irradiation was observed. It is worth mentioning that accelerated UV aging did not affect the antibacterial activity (toward *S. aureus*) of the EZ coating. Previous research [21] demonstrated that 24 h Q-SUN irradiation did not lead to a decrease in the antimicrobial properties of the coatings containing zinc oxide. These results confirmed that the shielding properties of ZnO nanoparticles were able to protect the activity of the EZ layer after 72 h of UV irradiation. Both 24 h and 72 h of Q-SUN irradiation had no effect on the activity of the E and EZ films against *E. coli*. Previous research [36] demonstrated that the antimicrobial activity against *E. coli* of the coating that contained plant extract was actually improved after Q-SUN irradiation. Moreover, Kairyte et. al. [37] confirmed that, zinc oxide nanoparticles prevented the inactivation of EZ coating after irradiation due to their UV-blocking properties.

**Figure 3:**
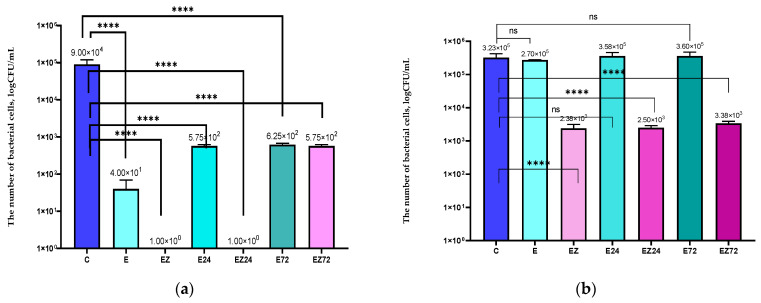
The influence of coatings on: (**a**) *P. syringae*, (**b**) *B. subtilis*; C—uncoated film; E—film coated with the HPMC layer with *Achillea millefolium* L., *Hippophae rhamnoides* L., and *Hypericum* L. extracts; EZ—film coated with the HPMC layer with *Achillea millefolium* L., *Hippophae rhamnoides* L., and *Hypericum* L. extracts and nano-ZnO; E24, E72 –blend film coated with E active layer after 24 h and 72 h of Q-SUN irradiation; EZ24, EZ72—blend film coated with EZ active layer after 24 h and 72 h of Q-SUN irradiation. One-way ANOVA; ****—*p* < −0.0001; ns (non-significant)—*p* > 0.5.

The films modified with the selected extracts were active against *Pseudomonas* sp. and *Bacillus* sp. [9,10,11,12,13,14,15,16,17,18]. However, the E layer did not inhibit the growth of *P. syringae* but decreased the number of cells that were viable. Moreover, the E sample neither inhibited the growth nor decreased the number of *B. subtilis* cells (Figure 3a,b), which means that the extracts’ presence in the HPMC carrier did not exhibit antibacterial properties against bacilli cells. Contrary findings were described in a previous study [7]. The HPMC modified with *Glycyrrhiza* L. and *Scutellaria baicalensis* solution inhibited *B. subtilis* cells completely. Higher activity was noted for the EZ coating (contained nano-ZnO). This material completely inhibited *P. syringae* growth and reduced the number of *B. sutilis* cells. The higher activity of the EZ in comparison to the E coating might have been caused by the synergism between active agents or by the higher grammage of EZ coating in comparison to the E layer. Sarhadi et. al. [38] confirmed that the addition of zinc oxide nanoparticles increased the thickness of the active layer, leading to an improvement in the antimicrobial properties of the films. As seen in Figure 3a,b, 24 h of accelerated UV aging did not affect the activity of the EZ coating against both strains. Nevertheless, it decreased the activity of the E layer toward *P. syringae*. Moreover, Q-SUN irradiation did not affect the activity of both modified films against *P. syringae* after 72 h of irradiation. However, it decreased the effectiveness of the E coating toward *B. subtilis*. These results confirmed that ZnO nanoparticles protected the antimicrobial activity of the EZ coating after 24 h (against both strains) but not after 72 h of UV treatment (toward *P. syringae*). Previous studies demonstrated that Q-SUN irradiation led to a decrease in the antibacterial effectiveness of the coating with ZnO nanoparticles against *P. aeruginosa* after 24 h [21]. Alternatively, the current findings indicate that the accelerated irradiation (for 1 day) did not affect the antimicrobial properties of the EZ coating. This could be explained by the presence of *Achillea millefolium* L., *Hippophae rhamnoides* L., and *Hypericum* extracts in the carrier, which are rich in compounds that could demonstrate UV irradiation barrier properties. This could also be due to the addition of nanoparticles of zinc oxide with UV-shielding properties. The difference in both activity and sensitivity of *P. aeruginosa* and *P. syringae* strains could be a reason for this phenomenon.

**Figure 4:**
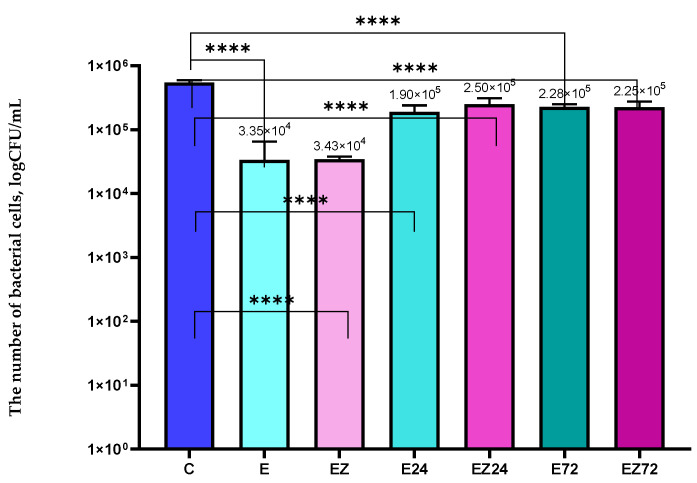
The influence of coatings on *C. albicans*. C—uncoated film; E —film modified with the HPMC layer with *Achillea millefolium* L., *Hippophae rhamnoides* L., and *Hypericum* L. *extracts*; EZ—the film coated with the HPMC layer with *Achillea millefolium* L., *Hippophae rhamnoides* L., and *Hypericum* L. extracts and ZnO nanoparticles; E24, E72—film coated with E layer after 24 h and 72 h of Q-SUN irradiation; EZ24, EZ72—film coated with EZ active layer after 24 h and 72 h of Q-SUN irradiation. One-way ANOVA; ****—*p* < −0.0001.

Although the results showed that films coated with E and EZ were not able to inhibit the *C. albicans* growth, they both significantly decreased the number of viable cells (Figure 4). The previous research demonstrated that active coatings with ZnO nanoparticles also decreased the number of *C. albicans* cells [21]. However, the coating with the nanofiller completely inhibited the growth of yeast [39]. The 24 h and 72 h of Q-SUN irradiation had a marked influence on the effectiveness of the E and EZ coatings against *C. albicans*, decreasing their activity. After the irradiation, the treated coatings had a fewer number of yeast cells, but their activity was lower than before the UV aging.

**Figure 5:**
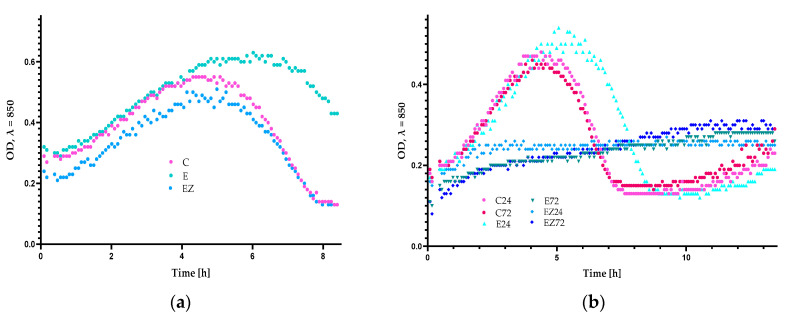
OD over time for the *P. syringae* after (**a**) 9 h and (**b**) 13 h of incubation; C—blend film; E—film coated with the HPMC with *Achillea millefolium* L., *Hippophae rhamnoides* L., and *Hypericum* L. *extracts*; EZ—the blend film coated with the HPMC with *Achillea millefolium* L., *Hippophae rhamnoides* L., and *Hypericum* L. extracts and ZnO nanoparticles; C24, C72—blend film after 24 h and 72 h of Q-SUN irradiation; E24, E72—film coated with E active layer after 24 h and 72 h of Q-SUN irradiation; EZ24, EZ72—film coated with EZ active layer after 24 h and 72 h of Q-SUN irradiation.

The results after the examination of antiviral properties demonstrated that the titer of the phi6 particles that were incubated with the uncoated film was 3.61 × 10^6^ PFU/mL. Analyzing the growth of the host with the phages for the sample described above, it was seen that after 5.5 h, a drop in OD was observed (Figure 5a). The results indicated that Φ6 eliminated most of the bacteria. The phages that were incubated with the EZ sample also led to a decrease in the growth rate of *P. syringae* after 5.5 h of incubation. Moreover, the titer of virus particles was 3.55 × 10^6^ PFU/mL. It was noted that the EZ-coated film was not active against phi6 phage.

An analysis of OD of the host cultivated with the Φ6 bacteriophages over time after their incubation with the E sample was also performed (Figure 5a), with the parameter’ fall observed after 7.5 h. Additionally, the titer of the phi6 lysate was significantly lower (9.79 × 10^5^ PFU/mL) than the titer observed for the unmodified film. These findings confirmed that the E sample exhibited slight activity against Φ6 bacteriophage, in contrast to noncovered film and EZ. Contrary results were observed in the previous research [39], which demonstrated that the coatings containing ZnO nanoparticles and *Formitopsis betulina*, *Verbascum* L., or *Uncaria tomentosa* extracts exhibited the complete reduction in the Φ6 lysate titer compared with the noncovered films. Moreover, the layers containing ZnO nanoparticles without plant extracts were not effective against the phi6 phage. This confirmed that *F. betulina*, *Verbascum* L., and *U. tomentosa* extracts were seen to be more active against viral particles than *Achillea millefolium* L., *Hippophae rham-noides* L., and *Hypericum* L. extracts. *Verbascum* L. exhibits antiviral properties [40,41] and its alcoholic extracts have even been used in therapy, improving the health of patients with COVID-19. Additionally, *F. betulina* and *U. tomentosa* extracts were also mentioned as antiviral materials [42,43,44].

The results of the antiviral analysis of the uncoated film (C) demonstrated that 24 h and 72 h irradiation (C24, C72) had no effect (Figure 5a,b) on the film with an observed lack of activity before and after accelerated UV aging. A drop in OD was observed after 4.5 h of incubation of *P. syringae* with phages. Furthermore, the titers of phage lysate observed in the case of irradiated film (24 and 72 h) were found to be insignificantly different: 3.58 × 10^6^ PFU/mL (24 h) and 3.56 × 10^6^ PFU/mL (72 h).

The results of the investigation of the influence of UV irradiation (24 h) on the antiviral activity of the E sample emphasized that the irradiation neither decreased nor increased the activity of the E-coated film (Figure 5b). Moreover, the differences between the titers observed for E coating pre- and post-irradiation for 24 h were statistically insignificant. Contrary results were observed for the E sample irradiated for 72 h where a drop in OD was not observed (Figure 5b). Furthermore, the bacteriophage titer was significantly lower (3.91 × 10^3^ PFU/mL), confirming the antiviral activity of the E coating after 72 h of UV treatment.

The investigation of the influence of Q-SUN irradiation (24 h and 72 h) on the antiviral properties of the EZ active coating, confirmed that accelerated irradiation improves the activity of the materials (Figure 5b). It should be noted that no drop in OD was observed for the host incubated with phages after their incubation with irradiated (for 24 h and 72 h) EZ sample. Moreover, the change in titers of the Φ6 lysates during the UV irradiation (3.74 × 10^3^ PFU/mL after 24 h and 3.44 × 10^3^ PFU/mL after 72 h) confirmed that EZ-coated film was more active after UV irradiation than nontreated material. Similar results were observed in previous studies [20,21]. The active layer, which was not effective before UV irradiation, demonstrated moderate antiviral activity after 24 h of Q-SUN irradiation. However, after 48 h of irradiation, the layer’s activity decreased. Mirzapoor et al. [45] noted that after the sunlight exposure of the coating with ZnO nanoparticles, the molecules of oxygen and water induce active agents called ROCs and VOCs. Due to their high reactivity, they could lead to the appearance of cavities in the virus envelopes and finally destroy them. It might be concluded that the active layers that contain zinc oxide nanoparticles may have been activated through Q-SUN irradiation. This led us to assume that not only nano ZnO but also the plant extracts have increased the antiviral activity of EZ coating (after 72 h of irradiation) due to both their synergistic effect and the shielding properties of nano-ZnO [21]. Additionally, based on the assumption that bacteriophage phi6 can act as a surrogate for the SARS-CoV-2 virus, it might be concluded that the coatings that are active against phi6 particles could also be effective against coronavirus [21,22].

### 3.2. SEM Analysis of Uncoated and Coated PLA/PHBV Films

**Figure 6:**
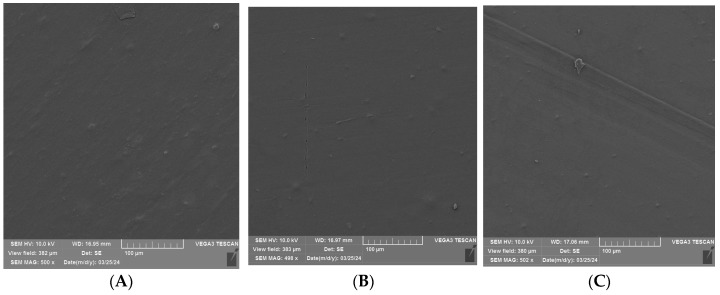
SEM micrographs of PLA/PHBV surface (**A**) before irradiation; (**B**) after 24 h of Q-SUN irradiation; (**C**) after 72 h of Q-SUN irradiation.

**Figure 7:**
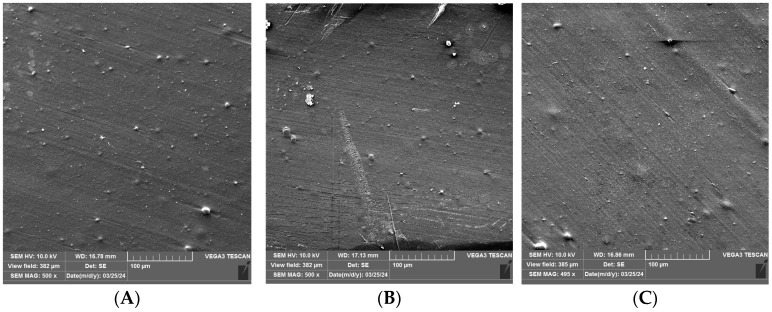
SEM micrographs of PLA/PHBV films coated with the E coating (**A**) before irradiation; (**B**) after 24 h of Q-SUN irradiation; (**C**) after 72 h of the Q-SUN irradiation.

**Figure 8:**
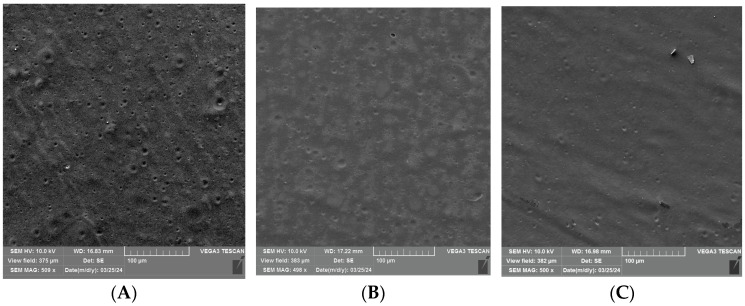
SEM micrographs of PLA/PHBV films coated with the EZ coating (**A**) before irradiation; (**B**) after 24 h of Q-SUN irradiation; (**C**) after 72 h of Q-SUN irradiation.

An SEM analysis was performed to visualize the surface of the unmodified and coated PLA/PHBV films before and after 24 h and 72 h of Q-SUN irradiation (Figure 6, Figure 7 and Figure 8). It was noted that 24 h of Q-SUN irradiation did not influence the sample surface as the differences between the samples irradiated for 24 h were not observed. Similar results were seen in previous studies [21,22]. The 48 h of Q-SUN irradiation did not influence the morphology of PLA/PHBV films. However, longer (72 h) accelerated irradiation slightly influenced the surface of the blends. It was demonstrated that the uncovered films had slightly rougher surface with some visible scratches after 72 h of irradiation. As one can notice in Figure 7A–C and Figure 8A–C, the active coatings were clearly visible on the film surface. In addition, the biopolymer blends were thoroughly and homogeneously covered with active antimicrobial layers. Similar observations have been made in a previous study [22] which clearly showed active homogenous layers, visible on the polypropylene surface. Furthermore, it was noted that the E coating was more homogenous than EZ coating. Additionally, spherical and convex particles were observed on the E layer before and after irradiation, which might have been caused by some contamination of the surface. There were also holes visible in the EZ coating, which could have been created during the drying process of coated surface. Carvalho et al. [46] mentioned that a thinner coating presented a more compact morphology in comparison to the thickest layers. The authors observed that an increase in coating thickness led to the formation of less compact layers; they also observed that the thinner coatings with nanoparticles of zinc oxide exhibited lower antibacterial activity than the thickest ones.

Surface analysis of the Q-SUN irradiation impact on the active coatings noted that 24 h and 72 h of UV-agin UV aging g had no significant influence on the morphology of the E coating (Figure 7B,C). Scratches were visible on the surface of both the irradiated and nonirradiated E layer. Moreover, small pores, holes, and breaks were not observed, even after 72 h of Q-SUN irradiation. Similar results were shown in a previous study [22]. The Q-SUN irradiation altered the surface morphology of the EZ layer (Figure 8A–C). There were fewer pores and holes in the coating after irradiation. In addition, 72 h of irradiation had a greater influence on the EZ coating than 1 day of UV aging. Longer irradiation led to higher homogeneity of the layer. Small pores and breaks observed on the EZ layer before and after 24 h irradiation (with Q-SUN) could have an influence on the release of active compounds from the antimicrobial coating. The active layer with pores and holes might, at the beginning, be more effective than the coating with a lower number of holes, although this might be for a shorter period of time.

### 3.3. Results of FTIR-ATR Analysis

**Figure 9:**
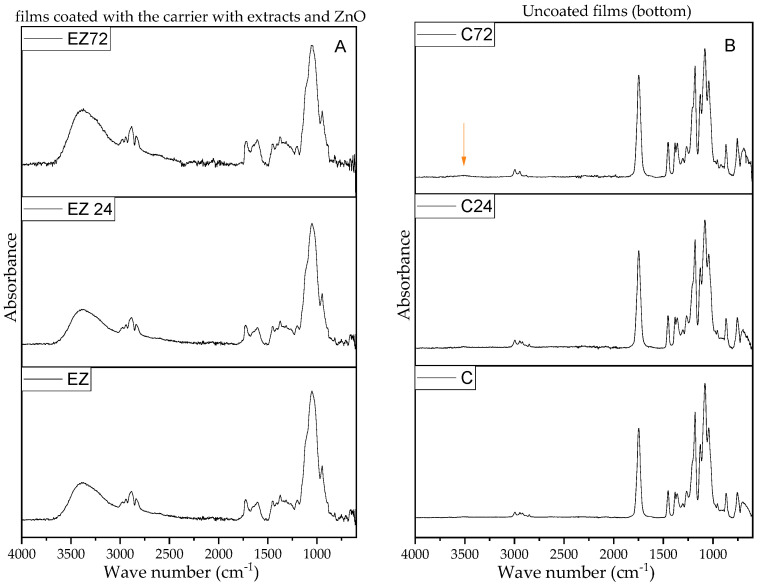

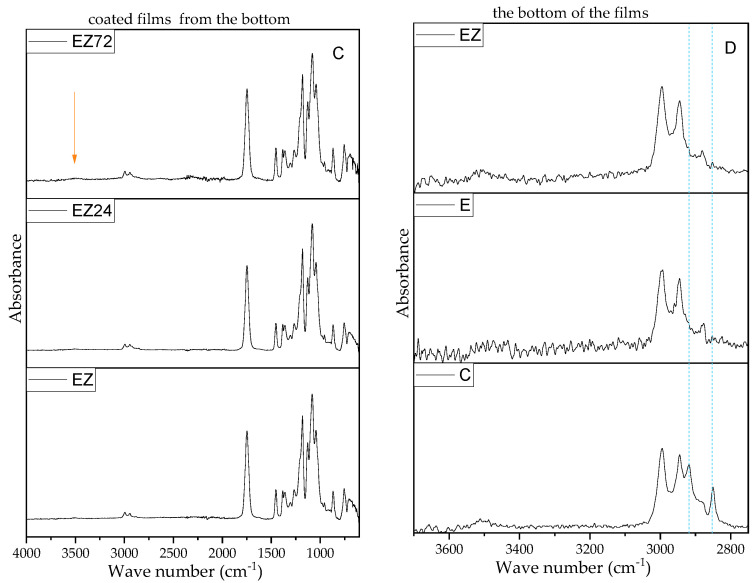
FTIR-ATR spectra of (**A**) selected EZ-coated film before and after UV treatment; (**B**) uncoated film before and after UV treatment; (**C**) EZ-coated film before and after UV treatment; (**D**) bottom (uncoated side) of the uncoated and coated films. The rest of the spectra are presented in the Supplementary Information.

Within this study, both sides of the films were analyzed using FTIR, pre- and post-UV treatment to investigate the influence of irradiation on the coating and the film. Figure S2A presents FTIR spectra for the different types of coatings, including pure carrier (HPMC) and the mixed systems with the extracts and composite additives. Comparing the pure coating and the mixed systems, it is clear that broadband ranging from 3648 to 3020 cm^−1^ assigned to axial stretching OH groups is shifted to lower wave numbers with a peak maximum (with higher intensity) at 3451.4 cm^−1^ and for HPMC to 3388.5 cm^−1^ in the case of the modified coatings. This could have been caused by internal H-bonding formation between the hydrocolloid carrier and the extract compounds. Additionally, the presence of additives can be confirmed with a small peak at 820 and 814 cm^−1^, for E and EZ, respectively, which do not exist in the pure HPMC coating. Comparing the E and EZ coatings, there are some shifts toward smaller wave numbers for coatings with nano-ZnO—from 1726 to 1720 cm^−1^ and 820 to 814 cm^−1^ which can indicate some interaction between extract components and ZnO nanoparticles. FTIR spectra in Figure 9A confirmed the presence of HPMC based on the sample surface [47]. In the case of both coatings (there were no significant differences pre- and post-UV irradiation (Figure 9A and Figure S2B), except a slightly higher intensity at a peak around 3648–3020 cm^−1,^, which might be caused by polysaccharide scission affected by the irradiation or a hydrolytic effect. However, these findings can be neglected due to any significant changes in the film’s appearance. Obtained FTIR spectra of modified films from the uncoated side are typical for PLA-based blends [48]. A sharp peak with high intensity at 1750 cm^−1^ confirmed the excess of PLA in the blend [49]. Comparing the unmodified surface of the films revealed a barely visible peak at 3520 cm^−1^ in samples irradiated for 72 h (Figure 9B, indicated by red arrow). The presence of OH groups could be an indication of the beginning of some kind of polyester degradation. The appearance of thissmall peak was observed for uncoated and coated samples, which indicated that the coating material did not fully protect the blend polyesters from UV irradiation (Figure 9B and Figure S2C). Interestingly, in the case of the coated films, two peaks at 2848 cm^−1^ (assigned to stretching vibration of methyl groups and 2921.5 cm^−1^ for C-H and CH_3_ groups of the side chains [50]) disappeared despite the fact that the coating was from the other side of the analyzed film (Figure 9C, indicated by dotted blue lines). This hard-to-explain phenomenon occurred regardless of the type of coating (HPMC and modified carrier) and without direct contact of the coating with the analyzed surface. In some studies related to modified polyesters, there were similar differences in the spectra [51,52]; however, only Valerini et al. attempted to explain this phenomenon in their work related to PLA coated with deposited ZnO [53]. They suggested the presence of some impurities in the polymer matrix. In our case, we have rejected this assumption due to the fact that both unmodified and coated films before and post-UV treatment, analyzed in a few random points, still showed these two peaks. We would like to suggest that the adhesion of the coating to the thin film or the presence of OH groups in its structure inhibited the oscillation of the methyl groups and that due to its origin from the side chain, the unmodified material can “move” more freely. Additionally, methyl groups in regions of ca. 2990–2850 cm^−1^ exhibited low polarity (barely visible) that could have been affected in some way by the HPMC groups; thus, low or barely visible absorption might be registered [54]. This phenomenon needs further examination.

### 3.4. CIELab Measurement Results

Table 1 shows the results of the color determination of unmodified and coated films before and after UV irradiation. The uncoated and nontreated PLA/PHBV film was used as a standard for the determination of color changes. As seen here, the pristine film was a highly transparent colorless material. After the application of the coating, it became more yellowish (higher b* value) with a green tint (a* toward minus values); however, coatings and irradiation did not affect the lightness (L*) of the samples. The changes are caused by the intensive greenish color of the extract, but due to the application of a thin layer of coating, it did not affect the film color dramatically. Comparing the parameters—color change (∆E), yellowish index (YI), and chroma (C*), which a related to the intensity of the color—it was noted that the values increased after UV treatment. The longer the period of UV irradiation, the higher the values of all parameters. Color changes decreased with the addition of ZnO to the coating composition, which is an advantage in the case of food packaging materials. The inhibiting activity of the nanofiller on color changes of a hybrid system based on extracts from cocoa bean shell waste and zinc oxide particles in the pectin matrix was also observed in other studies [55].

## 4. Conclusions

This study showed that the composite coatings applied directly to the polyester blend packaging film maintained their functionality after UV treatment. The results of the experiments determined that the E coating with the extract of *Achillea millefolium* L., *Hippophae rhamnoides* L., *and Hypericum* L., as well as the EZ coating, which contained the herb extracts, and the addition of ZnO nanoparticles inhibited *S. aureus* and *E. coli* growth completely. The EZ coating also inhibited *P. syringae* cells’ growth; however, the E coating only reduced their number. Both coated samples decreased the number of *B. subtilis* and *C albicans* cells, but they were not effective against phi6 phage particles. Our findings demonstrated that Q-SUN irradiation decreased the antibacterial activity of the E coating against *S. aureus*, *P. syringae*, and *C. albicans*. UV aging did not affect the antibacterial activity of the E layer against *E. coli* and *B. aubtilis*. However, it did improve its antiviral effectiveness. It was revealed that UV irradiation did not affect the antibacterial activity of the composite EZ layer against *S. aureus*, *E. coli*, and *B. subtilis* but decreased EZ effectiveness against *C. albicans*. Accelerated UV aging did not affect the activity of the EZ coating against *P. syringae* after 24 h of irradiation. Nevertheless, this deteriorated its antimicrobial properties against viable cells of *Pseudomonas*. In summary, the results of our work determined that 24 h, and even up to 72 h, of Q-SUN irradiation improved the antiviral effectiveness of the EZ layer. SEM analysis visualized PLA/PHBV blend films before and after 24 h and 72 h of Q-SUN irradiation. It was noted that Q-SUN irradiation did not significantly affect blend morphology. Moreover, biopolymer samples were thoroughly and homogeneously covered with antimicrobial layers. Additionally, differences between pure HPMC, E, and EZ coating morphologies were observed and indicated stronger bonding formation between ZnO nanoparticles and the extract components in the hydrocolloid carrier (shifts of the FTIR spectra peaks). It was also noted that 24 h and 72 h of UV aging had no significant influence on the morphology of the functional coatings; however, UV irradiation slightly affected the polyester structure (barely visible peak assigned to -OH groups). What is interesting is that FTIR analysis showed that the covered polyester material (the opposite side to the coating) altered the morphology or interaction between functional methyl groups, and this phenomenon requires further investigation. CIELab analysis results revealed that the applied coating slightly affected film color and nano-ZnO presence in the composite coating decreased color changes after UV irradiation.

## Figures and Tables

**Table 1:** CIELab scale color measurement results of the films.

Sample	L*	a*	b*	∆E	YI	C*
C	97.3 (0.04)	0.04 (0.01)	0.37 (0.05)	-	0.543	0.372
C24	97.3 (0.25)	0.06 (0.01)	0.34 (0.04)	0.036	0.499	0.345
C72	97.5 (0.06)	0.09 (0.02)	0.37 (0.04)	0.206	0.542	0.381
E	96.8 (0.09)	−0.15 (0.03)	1.81 (0.25)	1.532	2.739	1.866
E24	96.7 (0.15)	−0.18 (0.03)	2.41 (0.04)	2.090	3.553	2.417
E72	96.7 (0.04)	−0.29 (0.06)	2.62 (0.22)	2.367	3.951	2.695
EZ	96.8 (0.07)	−0.15 (0.01)	1.81 (0.07)	1.101	2.131	1.458
EZ24	96.9 (0.05)	−0.16 (0.03)	1.86 (0.13)	1.575	2.730	1.857
EZ72	97.0 (0.06)	−0.18 (0.03)	1.89 (0.21)	1.625	2.845	1.938

## Data Availability

Data are contained within the article.

## Supplementary Materials

The following supporting information can be downloaded at: https://www.mdpi.com/article/10.3390/ma17133048/s1.
