# Supplementary material for: The Influence of Functional Composite Coatings on the Properties of Polyester Films before and after Accelerated UV Aging
Source: Materials (Basel). 2024 Jun 21;17(13):3048. doi: 10.3390/ma17133048 (PMC11242206; doi:10.3390/ma17133048)
Supplement: Supplementary file 1 [file materials-17-03048-s001.zip › materials-2998178-supplementary.pdf]

SI - Supplementary Information

The Influence of Functional Composite Coatings on the Properties of Polyester Films before and after Accelerated UV Aging

Małgorzata Mizielińska, Magdalena Zdanowicz, Alicja Tarnowiecka-Kuca and Artur Bartkowiak *

Center of Bioimmobilisation and Innovative Packaging Materials, Faculty of Food Sciences and Fisheries, West Pomeranian University of Technology Szczecin, Janickiego 35, 71-270 Szczecin, Poland; mmizielinska@zut.edu.pl (M.M.); mzdanowicz@zut.edu.pl (M.Z.); alicja.tarnowiecka-kuca@zut.edu.pl (A.T.-K.)

* Correspondence: artur.bartkowiak@zut.edu.pl

Figure S1a demonstrated the growth of *Staphylococcus aureus* in rea-time. It was indicated that OD for the control sample (LB medium with *S. aureus* culture) increased after 22 h of incubation confirming that the bacterial cells were viable. It was noted that OD did not change for the A.m sample (10% *Achillea millefolium L.* extract in LB medium with *S. aureus* culture) even after 21 h of incubation. The result confirmed that the extract was active against studied microorganism. As was emphasized in Figure SI1a, OD fall was observed for: H.l (10% *Hypericum L* extract in LB medium with *S. aureus* culture); H.r (10% *Hippophae rhamnoides L.* extract in LB medium with *S. aureus* culture) and for the M (10% solution of 3 extracts in LB medium in wt ratio 1:1:1 with *S. aureus* culture). It means that these extracts and their solutions were influenced *S. aureus* growth which was found to be inhibited.

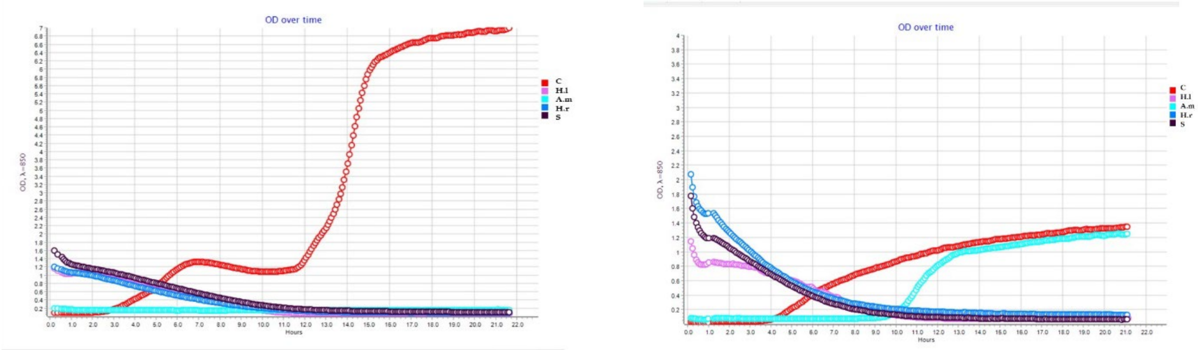

Figure S1a. OD over time for the *S. aureus* after 21 h of incubation. C—*S. aures*; H.l - *Hypericum L.*; A.m – *Achillea millefolium L.* extract, H.r – *Hippophae rhamnoides* L. extract, S – the solution of 3 herbs extracts.

Figure S1b showed the growth of *Escherichia coli* in real-time. It was noticed that OD for the control sample (LB medium with *E. coli* culture) increased after 21 h of incubation proving that the *E. coli* was viable. It was observed that OD increased for the A.m sample (10% *Achillea millefolium L.* extract in LB medium with *S. aureus* culture) after 12 h of incubation confirming that this extract was not active against Gram-negative bacteria. As was seen on Figure 1b, OD fall was observed for: H.l; H.r and M sample, confirming that the studied extracts and their solution were effective towards *E. coli* cells. The highest (however slightly) OD fall was noticed for the solution of all analyzed extracts (M). Based on these observations, the M solution was selected for the next tests.

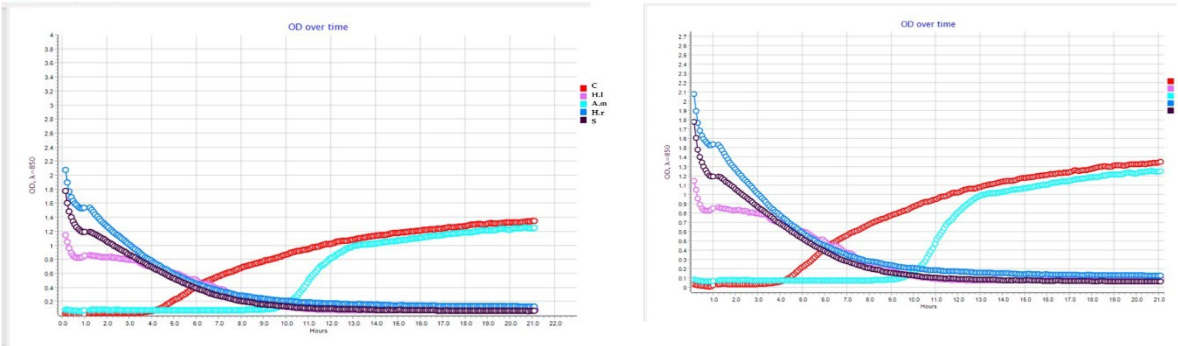

Figure S1 b. OD over time for the *E. coli* after 21 h of incubation. C—*E. coli*; H.l - *Hypericum L.*; A.m – *Achillea millefolium L.* extract, H.r – *Hippophae rhamnoides* L. extract, S – the solution of 3 herbs extracts.

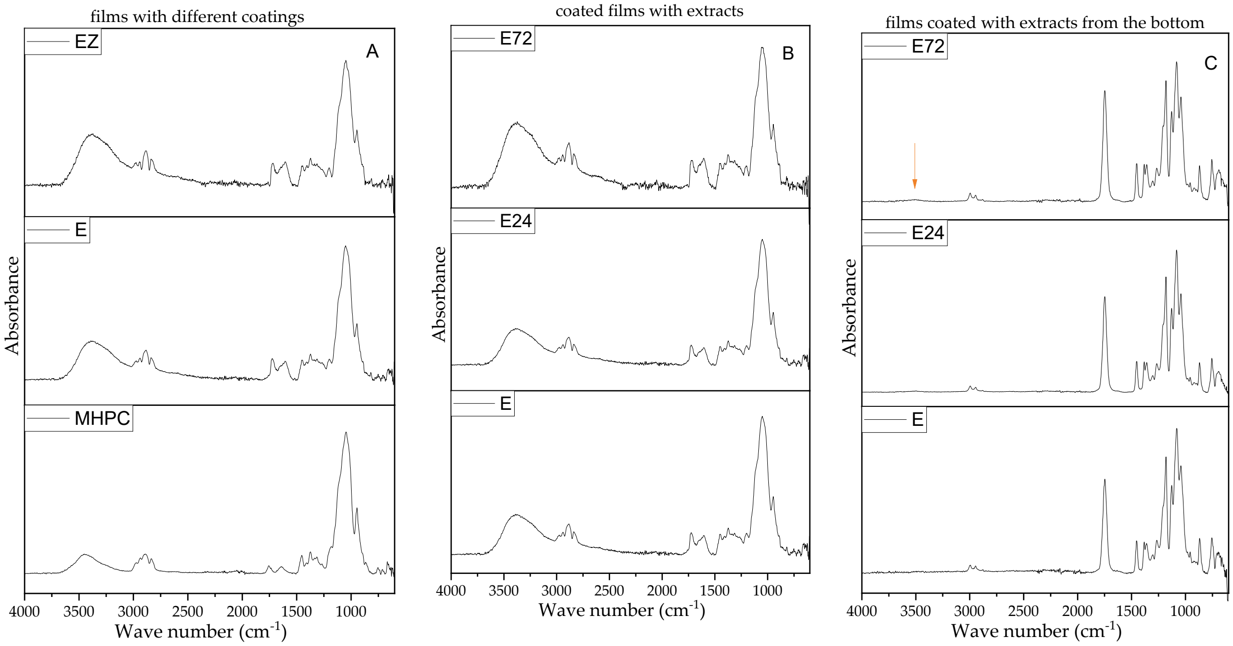

Figure S2. FTIR-ATR spectra of (A) different coatings on the polyester film (B) coated film w E system before and after UV treatment; (C) coated film E before and after UV treatment.
